# A Surprising Case of Triple Acute Hepatitis Infection

**DOI:** 10.3390/life13081761

**Published:** 2023-08-17

**Authors:** Ion Ștefan, Ioana-Mădălina Cristea, Alexia-Teodora Ștefan, Aurelian-Emil Ranetti, Carmen Adella Sirbu, Elena Rusu, Cosmin Alec Moldovan, Polliana M. Leru, Claudiu-Eduard Nistor

**Affiliations:** 1 Department of Infectious Diseases, ‘Dr. Carol Davila’ Central Military Emergency University Hospital, 134 Calea Plevnei, 010242 Bucharest, Romania; 2 Department of Medico-Surgical and Prophylactic Disciplines, Titu Maiorescu University, 040441 Bucharest, Romania; 3 Faculty of General Medicine, Carol Davila University of Medicine and Pharmacy, 020021 Bucharest, Romania; 4 Endocrinologic Department, Carol Davila University of Medicine and Pharmacy, 020021 Bucharest, Romania; 5 Clinical Neurosciences Department, Carol Davila University of Medicine and Pharmacy, 050474 Bucharest, Romania; sircar13@yahoo.com; 6 Academy of Romanian Scientists, 50044 Bucharest, Romania; 7 Department of Preclinical Disciplines, Titu Maiorescu University, 040441 Bucharest, Romania; elena.rusu@prof.utm.ro; 8 Clinical Department, Carol Davila University of Medicine and Pharmacy, 020021 Bucharest, Romania; 9 Colentina Clinical Hospital, 020125 Bucharest, Romania; 10 Thoracic Surgery Department, Carol Davila University of Medicine and Pharmacy, 020021 Bucharest, Romania

**Keywords:** acute hepatitis HAV, acute hepatitis HBV (AHB), acute exacerbation of chronic hepatitis B (AE-CHB), DNA HBV, acute hepatitis HEV

## Abstract

Viral hepatitis continues to be the leading cause of morbidity and mortality worldwide, but the burden has significantly diminished thanks to the large-scale use of vaccines and antivirals. However, there are still challenges regarding viral hepatitis management, especially when more than one pathogenic agent is involved. We present the case of a 45-year-old woman who had a simultaneous infection involving three hepatitis viruses: HAV, HBV, and HEV.

## 1. Introduction

Liver inflammation can be caused by a variety of triggers, especially five viruses, the hepatitis A virus (HAV), the hepatitis B virus (HBV), the hepatitis C virus (HCV), the hepatitis D virus (HDV), and the hepatitis E virus (HEV), which are well known for their hepatic tropism. Although remarkable progress has been made in better preventing, diagnosing, managing, and curing acute and chronic viral hepatitis, it continues to be a challenge for medical systems worldwide [1]. Each hepatic virus has its own specificity, and when it comes to acute viral hepatitis, it seems that HAV, HBV, and HEV are the most frequent etiologies [2,3,4].

### 1.1. Hepatitis A

HAV has a fecal–oral route of transmission through close contact with an infected individual or by ingestion of contaminated food or water [5]. Direct transmission usually occurs from children to their parents, in MSM (men who have sex with men) communities, or through intravenous drug use [6]. Indirect transmission, by ingesting contaminated water or produce (fruits, vegetables, or seafood), is also possible due to the increased resistance of the virus. HAV can survive high ambient temperatures and freezing [7].

Infections are reported all around the world, with an incidence in direct correlation with a person’s socio-economic status [8]. The highest prevalence of HAV infections is reported in developing regions like Sub-Saharan Africa and South Asia, and the lowest in economic strongpoints, such as the United States and Western Europe. There is an intermediate incidence in middle-income countries in Eastern Europe, North Africa, the Middle East, and Latin America [6].

A higher rate of HAV infections in developing countries means that the first contact with the virus happens at a younger age, resulting in frequent asymptomatic infections and a higher proportion of immunized adults. On the other hand, a lower incidence translates to a higher rate of unimmunized adults contracting the virus and developing symptomatic infections [6]. Thus, paradoxically, there is a higher prevalence of symptomatic infections in developed countries than in developing ones [8].

Usually, the hepatitis A virus enters the nonimmunized body through the digestive system and uses the hepato-enteric circulation to reach the liver. Here, it starts to replicate, and virions are detectable in the blood and stool before the onset of symptoms. Some days later, serum transaminase levels start to rise. The clinical phase starts a month after the first contact with the virus, and the individual experiences a range of symptoms: malaise, anorexia, nausea, vomiting, and fever. Adult infections are followed by diarrhea, jaundice, and hyperbilirubinemia. Typically, jaundice is the first symptom to resolve, and malaise and anorexia can last for months. The acute phase is followed by complete recovery and life-long immunization. Pediatric infections are usually inapparent [3,6,7].

An inactivated vaccine is available and seems to provide long-term protection [9]. A 2012 seroepidemiological study inquiring about 10 European countries found Romania to have an intermediate HAV endemicity and an incidence of 284/100,000 in the general population [10]. Acute HAV hepatitis could be prevented through vaccination, but in Romania, a nationwide vaccination program has not been implemented.

### 1.2. Hepatitis B

HBV is transmitted via infected blood, semen, and vaginal secretion. There are three main routes of transmission. The most common worldwide and typical for developing nations is vertical, from infected mothers to neonates. In developed nations, the most frequent route is the sexual one. At risk demographic categories are people with a high number of sexual partners and MSM. The third main route happens in healthcare settings through inadequate injections, blood transfusions, or dialysis. Other possible routes of transmission include nosocomial infections through contaminated instruments, organ donors, and close, non-sexual contact [11,12].

Hepatic B virus seems to have been in contact with approximately 30% of the world’s population, but the prevalence suffers wide variation and is correlated with the level of economic development [13]. The highest prevalence is reported in most of Africa, Southeast Asia, and parts of the Middle East, and in these regions, the infection typically occurs in the early stages, at birth, or during childhood. Intermediate prevalence occurs in eastern and southern Europe, Russia, and South America, and here, the infection can occur vertically, from mother to infant, or horizontally, from adult to adult. In low-endemic regions like North America, western Europe, and Japan, the main pattern of transmission is horizontal, usually occurring through unprotected sexual contact [13].

Once the hepatic B virus enters the unimmunized human organism, it infects and starts replicating in hepatocytes. Four to seven weeks after the infection, HBV DNA becomes detectable in the serum; it increases exponentially and then usually declines, preceding the onset of clinical hepatitis. HBV is not cytopathic; it does not inflict direct damage to the hepatocyte. The degree of hepatic injury and the clinical outcome of infection result from the interaction between the virus and the immune system of the host [14].

A robust immune response generates acute hepatitis B that is clinically recognizable by jaundice, malaise, and anorexia. It has a much higher rate of full clinical and serologic recovery. In rare cases, an excessive immune response causes fulminant HBV hepatitis with acute liver failure, a life-threatening condition. On the contrary, an overly permissive immune response ensures the persistence and continuous replication of virions in hepatocytes beyond 6 months and defines the progression into the chronic phase of the HBV infection. Usually asymptomatic, chronic liver inflammation can develop into cirrhosis and hepatocellular carcinoma [14,15]. Moreover, the inadequate cellular immune response can generate extrahepatic events: glomerulonephritis and vasculitis. A small percentage of people living with chronic hepatitis B are cured spontaneously [11,16].

Notably, children have a smaller rate of symptomatic acute hepatitis, and they more frequently develop chronic hepatitis [11].

HBV infection can be prevented by immunization. A safe and effective monovalent vaccine made from recombined DNA is available, and most European countries have made it a part of the childhood immunization scheme [12]. Romania’s population has benefited from a nationwide vaccination program against HBV since 1995.

### 1.3. Hepatitis E

HEV has a wide animal reservoir. Its main route of transmission is zoonotic, from infected pigs, wild boar, and deer to humans ingesting uncooked meat products. Once infected, pigs are asymptomatic and shed a large number of viruses in the stool, thus contaminating water sources. Consequently, HEV has been found in fresh produce and shellfish [5]. It can also be transmitted vertically, from mother to neonate, and horizontally, especially in MSM communities, through the transfusion of contaminated blood [2].

As a fairly recently discovered pathogen, its epidemiological patterns are still a puzzle. The incidence of HEV infections varies between and within regions and overtime, for yet unclear reasons [17].

The main target of the hepatic E virus is the hepatocyte, where it can replicate. After an incubation period of 2 to 9 weeks, HEV RNA is detectable in the blood and stool. The vast majority of infections in immunocompetent hosts are asymptomatic, but when symptoms do occur, they usually happen a few days after the RNA becomes detectable. During this acute phase, the patient develops unspecific symptoms like fatigue, anorexia, and nausea, and elevated liver enzymes are found. Fulminant hepatitis is extremely rare. Most cases of infection resolve spontaneously by acquiring an unsterilizing immunity—a reinfection is possible [17,18].

The acute phase is of particular concern in immunocompromised patients, like organ transplant recipients and those suffering from hematological disease or untreated VIH infection. Moreover, this category of patients cannot mount an efficient immune response, so the viral multiplication continues. If the HBE RNA is still detectable 6 months after the first positive result, chronic hepatitis E is diagnosed. The majority of patients are asymptomatic, but some can experience extrahepatic manifestations like neurologic amyotrophy, Guillain–Barré syndrome, or glomerulonephritis. Within a 3 to 5 years span, they develop liver fibrosis and cirrhosis [17,18,19].

A vaccine for preventing acute hepatitis E was licensed in China in 2011. This vaccine does not offer complete protection; sub-clinical infections can still occur. Furthermore, the long-term effects of this product are still undocumented [20].

## 2. Case Report

We present the case of a 45-year-old Caucasian woman diagnosed with mild iron deficiency anemia due to menstruation losses in treatment with iron tablets who presented to the emergency room with jaundice, nausea, myalgia, and fatigue.

The patient lived in an urban area in Romania during the COVID-19 pandemic, has a desk job, and no other household member displayed any similar manifestations. A detailed medical history revealed that these symptoms were 8 days old and initially attributed to COVID-19. Despite numerous SARS-CoV-2 PCRs being negative, treatment with Azithromycin and NSAID (Piroxicam) was prescribed. Without any clinical improvement, the patient came to our clinic for diagnosis and treatment. Physical examination showed intense yellow-lemon jaundice, a discreet hepatomegaly, normal stools, and dark urine.

Extensive blood work was carried out. The immediate results exposed severe hepatitis (ALAT 3740 U/L, ASAT 1978 U/L, INR 1.72, prothrombin activity 54%) accompanied by adjacent hyperbilirubinemia (5-fold increase in total bilirubin with a predominance of the conjugated one). The patient was admitted to the Infectious Disease ward for further investigations and medical care. We ran an extensive serologic screening panel for acute hepatitis, including all hepatitic viruses (A, B, C, D, and E), VIH, CMV, Epstein–Barr Virus, leptospirosis, and COVID-19. It came back positive for HAV (IgM reactive), HEV (IgM = 45.2 U/mL), and HBV (Ag HBs reactive and HBc IgM reactive). These results called for a second serologic work-up in a different laboratory with added HBV DNA detection and quantification by Real-Time PCR. The results were identical, and DNA HBV had a value of 60,400 UI/mL.

Abdominal ultrasound showed hepato-splenomegaly and no biliary tract obstruction.

**Figure 1:**
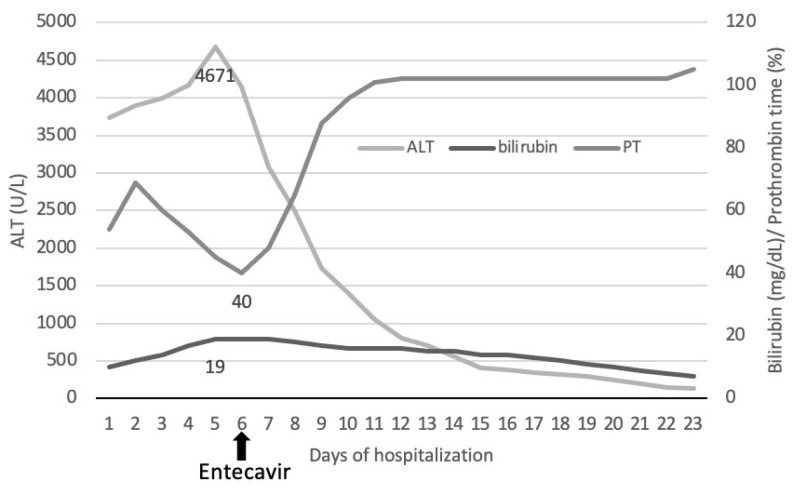
Hepatic function during hospitalization. An efficient response is observed after treatment with an antiviral molecule. Note: adapted from management of severe acute to fulminant hepatitis B: to treat or not to treat, or when to treat? Tillman et al., 2011, Liver International.

The patient received IV vitamin K, fresh plasma, essential amino acids, and albumin with clinical stagnation and paraclinical worsening liver inflammation (increasing ALAT, ASAT, bilirubin, hypofibrinogenemia, and prothrombin activity at 40%). At this point, we started therapy with nucleoside inhibitors of the HBV polymerase (a 0.5 mg daily dose of Entecavir), which was associated with a clear improvement in both clinical and paraclinical domains (Figure 1). Ten days later, the patient was discharged from the hospital, and treatment with Entecavir was maintained.

A medical visit 23 days after discharge revealed abolition of symptoms, a normal functioning liver (ASAT, ALAT, coagulogram, and fibrinogen within limits), and a serology with dynamic changes—HAV in the clearance process with positive IgM and IgG, HEV with negative IgM, and suppression of HBV replication with a DNA HBV < 10 UI/mL during Entecavir therapy, showing signs of seroconversion with a negative hepatitis B surface antigen, but with HBs antibodies in low quantity (0.31 mUI/mL). The blood work was repeated 3 weeks later with similar results except got a slight increase in HB antibodies (2.28 mUI/mL).

Lab tests 6 weeks later revealed a serologic surprise: undetectable Ag HBs coupled with a protective titer of HB antibodies (22.04 mUI/mL), all backed up by an undetectable DNA HBV. The patient was advised to continue treatment with Entecavir for 2 more months before reevaluation.

**Figure 2:**
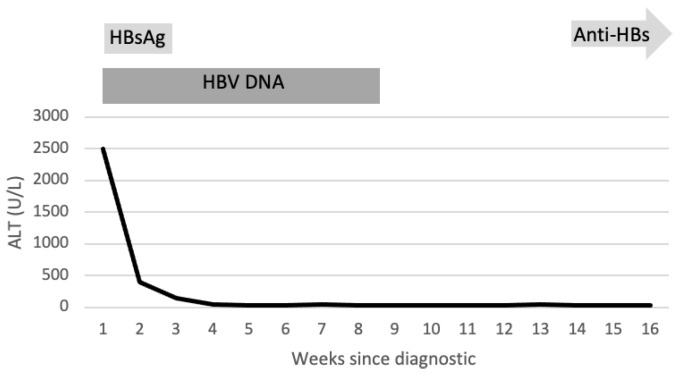
Resolution of acute hepatitis B during antiviral treatment started on the 6th day since diagnostic. Note: adapted from hepatitis B virus infection Trépo et al., 2014, Lance.

Two weeks after stopping Entecavir therapy, the blood work confirmed a sustained virological response with undetectable DNA HBV and AgHbs and protective HB antibodies (Figure 2).

## 3. Discussion

### 3.1. Diagnosis of Hepatitis E

Hepatitis E is a relatively new medical entity, and extended laboratory diagnosis tools are not available worldwide. For confirming acute HEV hepatitis, the European Center for Disease Control (ECDC) requires at least a serological assay with a positive IgM and rising IgG titer or a positive PCR [21]. The main concern with isolated IgM detection is its cross-reactivity with HAV, Epstein–Barr virus, and Cytomegalovirus, and the consequent false positivity [22,23,24]. In immuno-competent patients, it is mostly a diagnosis challenge without damaging the patient’s care since no etiologic treatments are available.

In our hospital setting, IgM HEV was the only diagnostic tool accessible, and, although positive, it was not enough to ascertain a diagnosis given its limitations.

### 3.2. Dynamics of Hepatitis B Markers

Hepatitis B infection can be confirmed and observed through its clinical phases by studying the kinetics of some serologic markers: HBV surface antigen (HBs Ag) and its antibody (HBs Ac), anti-HBc antibodies (IgM and IgG), and HBV “e” antigen (HBe Ag) with a paired antibody (HBe Ac). The HBV DNA detectable level is another confirmatory proof of hepatitis B infection. Thanks to these lab tests being widely available, HBV infection can be easily confirmed.

The challenge rests in distinguishing between two clinically similar conditions: acute hepatitis B (AHB) and an acute exacerbation of chronic hepatitis B (AE-CHB). Various studies have attempted to find significant differences between the two, looking into the medical history, symptoms, liver function tests, serologic markers, HBV DNA, and hepatic elastography [25,26,27]. It seems that there is no foolproof way to ascertain if a confirmed case of acute hepatitis B is the consequence of initial exposure to HBV or of a flare-up in chronic occult hepatitis B, but a few clues can be sought.

A detailed anamnesis is a good starting point, revealing old or new hazardous exposure to HBV (blood transfusions, medical procedures, unsafe sexual intercourse, etc.). Clinical signs of portal hypertension may suggest AE-CHB, but the signs and symptoms are similar for viral acute hepatitis [16]. Laboratory tests of liver function, like transaminases, prothrombin time, albumin, and bilirubin serum levels, are pathological in both AHB and AE-CHB and hold no discriminatory value [28]. IgM anti-HBc serum presence is a well-known indicator of AHB, but it has been reported that these antibodies are also produced, in lower titers, in AE-CHB, and more recent, sensitive qualitative test are able to detect it [29,30]. This can cause misdiagnosis, and so its diagnostic value is impaired. HBs Ag and HBe Ag serum levels seem to have a differently patterned evolution in AHB and AE-HBC, though they lack standardization for day-to-day medical practice [17]. A valuable clue can arise from initial DNA HBV levels, which are lower in acute hepatitis B [20].

Our patient lives in a country with an HBV infection incidence of 1.5–4.4/100,000, has no prior HBV vaccination, and has no family or personal history of acute or chronic hepatitis. Upon ER presentation, the blood work revealed positive HBs Ag, anti-HBc IgM, and 60,400 UI/mL of DNA HBV. HBe Ag, although an important serological marker, was unfortunately not performed. Based on these parameters, we diagnosed the patient with acute hepatitis B. The dynamic profile of these parameters cannot be reliable for distinguishing between acute hepatitis B and an acute exacerbation of chronic/inactive hepatitis B because the natural course was impaired by antiviral treatment.

### 3.3. Antiviral Treatment in Acute Settings

Large double-blind studies for direct antiviral treatment in acute hepatitis B are missing, making it an “off-label” recommendation. There are two main concerns with antiviral prescription in the acute phase: the virus’s high rate of spontaneous clearance (up to 95% reported in some studies) and the theoretical assumption that it inhibits antibody production. Despite this, direct antivirals have been used in practice as rescue therapy in severe acute hepatitis B, with promising preliminary results [31,32].

As acute hepatitis E usually runs a self-limited course, no antiviral treatment is needed in most cases. However, exceptionally, liver failure does occur in some patients, and there have been a few case reports of ribavirin being used as a salvage treatment [17].

No direct-acting antivirals are commercially available for HAV, but interferon and other antivirals, such as ribavirin and sofosbuvir, were reportedly used in a limited series of case reports [2,18].

We started Entecavir therapy when the paraclinical results reached an all-time low: prothrombin of 40%, increasing hypofibrinogenemia and lower transaminase values compared to previous days. We noticed an immediate, spectacular improvement and, in the long run, a consistent HBV clearance.

### 3.4. Ways of Infection and Possible Sources of Triple Infection

The hepatotropic viruses have a human and an animal reservoir with well-known modes of transmission: fecal–oral or through bodily fluids (blood, semen, and vaginal secretions). Some of them have common routes, and so dual infections are often described: HAV and HEV in food or water-borne outbreaks or through oral or anal intercourse, and HBV plus HCV hepatitis through sexual relations or procedures involving unscreened blood [5,33].

A triple infection is exceptional, and, to our knowledge, only one case has been reported so far. Butt et al. described a pediatric acute exacerbation of chronic HBV hepatitis due to an HAV and HEV coinfection [34]. No supposition about how the patient contracted the infectious agents is made.

With no scientific data to refer to, we relied on the biologic markers and a thorough anamnesis to identify the source or sources of infection in our patient. Starting with the biological proofs, we formed two hypotheses: an acute exacerbation of chronic HBV hepatitis or an acute triple viral hepatitis. The anamnesis revealed no reported outbreaks or other cases of acute illness in our patient’s social circle, despite all meals being shared with her family, so we found the first supposition unlikely. As for the second hypothesis, the only route of transmission that the hepatitis viruses A, E, and B share is the sexual one. Unfortunately, our patient was unwilling to disclose her sexual history, so we cannot confirm this theory, but we believe this to be the route of infection.

## Data Availability

No new data were created or analyzed in this study. Data sharing is not applicable to this article.
